# Are there independent predisposing factors for postoperative infections following open heart surgery?

**DOI:** 10.1186/1749-8090-6-151

**Published:** 2011-11-14

**Authors:** Ioanna Lola, Stamatina Levidiotou, Anastasios Petrou, Helen Arnaoutoglou, Efstratios Apostolakis, George S Papadopoulos

**Affiliations:** 1 Laboratory of Microbiology, University Hospital of Ioannina, Ioannina, Greece; 2 Dpt of Anesthesia and Postoperative Intensive Care, University Hospital of Ioannina, Ioannina, Greece; 3 Dpt of Cardiothoracic Surgery, University Hospital of Ioannina, Ioannina, Greece

**Keywords:** cardiopulmonary bypass, cardiac operations, open heart surgery, coronary artery bypass grafting, post-cardiac surgery infections, ICU infections, ICU-flora, MRSA

## Abstract

**Background:**

Nosocomial infections after cardiac surgery represent serious complications associated with substantial morbidity, mortality and economic burden. This study was undertaken to evaluate the frequency, characteristics, and risk factors of microbiologically documented nosocomial infections after cardiac surgery in a Cardio-Vascular Intensive Care Unit (CVICU).

**Methods:**

All patients who underwent open heart surgery between May 2006 and March 2008 were enrolled in this prospective study. Pre-, intra- and postoperative variables were collected and examined as possible risk factors for development of nosocomial infections. The diagnosis of infection was always microbiologically confirmed.

**Results:**

Infection occurred in 24 of 172 patients (13.95%). Out of 172 patients, 8 patients (4.65%) had superficial wound infection at the sternotomy site, 5 patients (2.9%) had central venous catheter infection, 4 patients (2.32%) had pneumonia, 9 patients (5.23%) had bacteremia, one patient (0.58%) had mediastinitis, one (0.58%) had harvest surgical site infection, one (0.58%) had urinary tract infection, and another one patient (0.58%) had other major infection. The mortality rate was 25% among the patients with infection and 3.48% among all patients who underwent cardiac surgery compared with 5.4% of patients who did not develop early postoperative infection after cardiac surgery. Culture results demonstrated equal frequencies of gram-positive cocci and gram-negative bacteria. A backward stepwise multivariable logistic regression model analysis identified diabetes mellitus (OR 5.92, CI 1.56 to 22.42, p = 0.009), duration of mechanical ventilation (OR 1.30, CI 1.005 to 1.69, p = 0.046), development of severe complications in the CICU (OR 18.66, CI 3.36 to 103.61, p = 0.001) and re-admission to the CVICU (OR 8.59, CI 2.02 to 36.45, p = 0.004) as independent risk factors associated with development of nosocomial infection after cardiac surgery.

**Conclusions:**

We concluded that diabetes mellitus, the duration of mechanical ventilation, the presence of complications irrelevant to the infection during CVICU stay and CVICU re-admission are independent risk factors for the development of postoperative infection in cardiac surgery patients.

## Introduction

Postoperative nosocomial infections are a considerable problem in cardiac surgery patients associated with prolonged hospitalization [1,2], increased short and long-term mortality [2,3] and augmented total treatment cost [4]. The proportion of coronary artery bypass patients at high-risk for infection is increasing because of the aging population, a growing number of patients undergoing "redo" procedures, and the frequency of conditions conferring both cardiovascular and infectious risks (obesity, diabetes mellitus) among this population [1].

The diagnosis of postoperative infection is sometimes difficult, since clinical and laboratory signs of inflammation may be caused not only by infection, but also by tissue injury and mainly by the systemic inflammatory response syndrome (SIRS) associated with cardiopulmonary bypass [5]. In addition, surgical patients usually receive systemic antibiotics, especially in the ICU, thus negatively influencing blood culture yield [5,6].

The aim of this study was to examine the incidence of early postoperative infections in patients undergoing on pump coronary artery bypass grafting (CABG) surgery, to analyze the kind of infection and the responsible pathogens as well as to identify the contributing risk factors for these infections.

## Materials and methods

This prospective study was conducted between May 2006 and March 2008 at the Cardio-Vascular Intensive Care Unit (CVICU) of University Hospital of Ioannina. Following ethical committee approval, all patients included consented to the terms of this study during the preoperative evaluation and discussion with the attending anesthesiologist.

The study included patients with coronary artery disease who underwent on-pump coronary artery bypass grafting (CABG).

Exclusion criteria were:

- Any sign of clinical infection preoperatively (fever of ≥38°C or elevated white blood cell count or elevated CRP).

- Preoperative mechanical ventilation for any reason.

- Preoperative hospital length of stay >3 days.

- History of: Ca, recent infection or fever of unknown origin, chronic benign disease, such as rheumatoid arthritis, systemic lupus erythematosus, and chronic administration of corticosteroids.

- Intraoperative exclusion criteria: accidental potential infection (e.g. accidental abolition of the sterile conditions of the surgical field), evidence of line's infection (tenderness, erythema or induration of the catheter exit site).

- Postoperative exclusion criteria: low cardiac output postoperatively observed by using continuous cardiac output monitoring. These patients were excluded, as they present a higher risk of acquiring an infection due to the fact that excessive manipulations are needed (infusion of fluids, inotropic support, intra-aortic balloon pump) and that host defenses may be impaired.

Patients' data that were routinely recorded preoperatively were as follows: age, height, weight, body mass index, body surface area, co-morbidities (see Table 1), hematological and biochemical laboratory results. Coronary angiography data, the presence of indwelling urinary catheter before the operation and the time of insertion, the presence of vascular catheters and the time of insertion and the presence of intra-aortic balloon counter-pulsation device (Intra-aortic balloon pump - IABP) were also recorded. Taking into account their underlying diseases, active conditions and co-morbidities, the Euroscore index was calculated accordingly. According to their Euroscore index, patients were divided in 4 groups: Group I: Euroscore index 1-2, Group II: Euroscore index 3-5, Group III: Euroscore index 6-12, Group IV: Euroscore index >12.

**Table 1:** Patients' characteristics.

Characteristic	Patients Ν	Percentage (%)
Male	133	77.3%
Female	39	22.7%
Diabetes mellitus	59	34.3%
Arterial hypertension	118	68.6%
Chronic obstructive pulmonary disease	22	12.8%
Chronic renal failure	11	6.4%
Peripheral vascular disease	23	13.4%
Stroke	19	11%
Unstable angina	35	20.3%
Recent myocardial infarction (within 90 days)	46	26.7%
Older myocardial infarction	31	18%
Pulmonary hypertension	2	1.2%
Hyperlipidemia	45	26.2%
Smoking	69	40.1%
Alcohol abuse	13	7.6%

Intraoperative and postoperative data were recorded to determine their influence on the development of infection (see Table 2).

**Table 2:** Intraoperative and postoperative data, perioperative transfusion.

Intraoperative and postoperative data	Values
**Intraoperative factors**	
Duration of anesthesia (hours)	6,35 ± 1,69
Duration of extra-corporeal circulation (min)	146.94 ± 55.14
Ischemia time (min)	109.27 ± 45.97
Degree of hypothermia (^ο^C)	30.27 ± 1.61
Emergency operation	11 (6.4%)
**Postoperative factors**	
Intra-aortic balloon pump (IABP)	18 (10.5%)
Duration of mechanical ventilation (days)	1.9 ± 4.23
Re-intubation	22 (12.8%)
Tracheotomy	8 (4.7%)
Enteral nutrition for >10 days	5 (2.9%)
Parenteral nutrition for >10 days	6 (3.5%)
Re-operation	13 (7.6%)
Complications in the CVICU	44 (25.6%)
Re-admission to the CVICU	20 (11.6%)
Length of stay in the CVICU (days)	4.23 ± 6.4
**Perioperative transfusions**	
N of RBC units transfused	4.37 ± 2.9
N of FFP units transfused	4.36 ± 3.8
N of PLT units transfused	5.85 ± 5.1

CVICU: cardio-vascular intensive care unit

Antibiotic prophylaxis with cefotaxime was administered routinely to all patients intravenously as a single dose (1g) at the induction of anesthesia and postoperatively 1g every 8 hrs for 3 days. In case of suspected infection, empirical antibiotic therapy was initiated. Treatment was modified according to culture results.

Diabetic patients received perioperatively continuous insulin infusion to maintain blood glucose levels between 110 and 150 mg/dL.

All patients included in the study were daily examined, during the morning and afternoon rounds, by the CVICU attending anesthesiologists for possible signs of infection. Hematological and biochemical tests, and chest x-ray were performed every day and vital data were recorded routinely every hour. Sternotomy site and indwelling catheters' insertion sites were examined, cleaned and properly dressed. Bronchial secretions were collected from all patients for culture before removing the endotracheal tube. Blood cultures from two peripheral sites, samples for cultures from urine and the tips of removed catheters were taken from all patients routinely. When wound infection was suspected, cultures were taken from the inner surface of the wound. In case of suspected infection, additional cultures of blood, urine, bronchial secretions or bronchoscopy brush samples and tips of removed arterial or venous catheters were ordered. The definitions of nosocomial infections were based on those proposed by the Centers for Disease Control and Prevention (CDC) [7].

Statistical analysis was performed using SPSS v 15.0. Results are presented as Mean values  ± Standard Deviation (SD). Statistically significant factors were initially identified at p < 0.20. These were used in a logistic regression model. Significance was then set at p < 0.10 and the factors were reduced with the Backward LR method. Final pruning of the model was carried out with forward LR logistic regression at p < 0.05 and interactions between statistically significant factors were also considered.

## Results

The study included 172 patients (77.3% males) of mean (±SD) age 66 ± 10 years old (range 36-85 years). Euroscore Group I (index 1-2) included 31 patients (18%), Group II (index 3-5) included 74 (43%), Group III (index 6-12) included 41 patients (24%) and Group IV (index > 12) included 26 (15%). Groups III and IV included 67 patients in total (39%) with a combined, estimated risk of death at 13% (12.61 ± 7.56%). Patients' characteristics are presented in Table 1.

Intraoperative and postoperative data as well as perioperative transfusions are shown in Table 2.

The duration of mechanical ventilation in the high risk groups III and IV (67 patients) was 2.79 ± 6.44 days and the length of stay in the CVICU was 5.85 ± 8.92 days. Eleven patients (16.4%) classified in the high risk group were readmitted to the CVICU, while five of them were readmitted twice. The reasons for readmission were respiratory (respiratory insufficiency due to pneumonia 3 pts, pleural effusion 2 pts, marked atelektasis 2 pts), cardiac (cardiac arrest 2 pts, intractable arrhythmia 1 pts), cerebrovascular accident (3 pts) and re-operation for a sternal wound debridement (3 pts).

Twenty four patients presented microbiologically documented nosocomial infection (n = 24, 13.95%). Six of these patients presented multiple infections. The sites of infections are presented in Table 3. Thirteen patients with infection died. In 6 of them, infection was considered the main cause of death while in the remaining 7 patients, infection contributed to death as a secondary cause on top of other complications, irrelevant to infection. Four out of those six patients were classified to the high risk groups of III and IV of the Euroscore scale. Therefore total mortality attributed to infection was 3.48% (6/172 pts) and mortality among infected patients was 25% (6/24 pts).

**Table 3:** Site of infections, number, percentage and incidence of infections in the studied 172 patients.

Site of infection	N, %	Incidence
Bacteremia	9 (30%)	5.23%
Sternotomy site infection	8 (26.7%)	4.65%
Infection of vascular catheters	5 (16.7%)	2.90%
Pneumonia	4 (13.3%)	2.32%
Mediastinitis	1 (3.3%)	0.58%
Surgical site infection, other than sternotomy	1 (3.3%)	0.58%
Infection of the intra-aortic balloon pump (IABP)	1 (3.3%)	0.58%
Urinary tract infection	1 (3.3%)	0.58%
**Total**	30 (100%)	17.42%

Causative microorganisms that were isolated and their resistance phenotype are presented in Table 4 while Table 5 presents the pathogens and their respective resistance phenotype per infection site. Gram-positive and gram-negative microorganisms were isolated in equal number of cases. Gram-positive cocci were the most frequent bacterial species isolated in patients with infection at the sternotomy and venous graft harvesting sites and in patients with mediastinitis, whereas gram-negative bacteria were the responsible pathogens in those with pneumonia and urinary tract infections and also the most common pathogens in patients with bacteremia. *Staphylococci *were isolated in 40% of cases (12 of the 30), 83.3% of which were coagulase-negative and 58.3% were resistant to methicillin. *Acinetobacter baumannii *was the predominant pathogen among gram-negative bacteria (26.7%), while 60% of enteric gram-negative bacilli were confirmed to produce ESBL (extended-spectrum β-lactamase).

**Table 4:** Isolated pathogens and their resistance phenotype.

Type of microorganism	Ν, (%)	Number of isolates/Resistance phenotype
**Gram-positive cocci**		
*Staphylococcus epidermidis*	8 (26.7%)	4/MRSE, 4/MSSE
*Staphylococcus aureus*	2 (6.7%)	1/MRSA, 1/MSSA
*Staphylococcus haemolyticus*	2 (6.7%)	2/MRSH
*Enterococcus faecium*	2 (6.7%)	2/VSE
*Enterococcus faecalis*	1 (3.3%)	1/VSE
**Gram-negative bacteria**		
*Acinetobacter baumannii*	8 (26.7%)	8/MBL(-)
*Pseudomonas aeruginosa*	2 (6.7%)	2/MBL(-)
*Escherichia coli*	3 (10%)	1/ESBL(-), 2/ESBL(+)
*Klebsiella pneumoniae*	1 (3.3%)	1/ESBL(+)
*Enterobacter cloacae*	1 (3.3%)	1/ESBL(-)
**Total**	30 (100%)	

MRSE: methicillin-resistant *S. epidermidis*; MSSE: methicillin-susceptible *S. epidermidis; *MRSA: methicillin-resistant *S. aureus; *MSSA: methicillin-susceptible *S. aureus; *MRSH: methicillin-resistant *S. haemolyticus; *VSE: vancomycin-susceptible *Enterococcus*; ESBL: extended-spectrum β-lactamase; MBL: metallo-β-lactamase.

**Table 5:** Type of microorganisms per infection site and their respective resistance phenotype.

Site of infection	Type of microorganism	Infections Ν	Number of isolates/Resistance phenotype
**Pneumonia**	*Acinetobacter baumannii*	3	3/MBL(-)
	*Pseudomonas aeruginosa*	1	1/MBL(-)
**Sternotomy site infection**	*Staphylococcus epidermidis*	5	3/MRSE, 2/MSSE
	*Enterococcus faecium*	1	1/VSE
	*Pseudomonas aeruginosa*	1	1/MBL(-)
	*Acinetobacter baumannii*	1	1/MBL(-)
**Mediastinitis**	*Staphylococcus aureus*	1	1/MRSA
**Surgical site infection other than sternotomy**	*Staphylococcus aureus*	1	1/MSSA
**Bacteremia**	*Staphylococcus epidermidis*	2	1/MRSE, 1/MSSE
	*Staphylococcus haemolyticus*	1	1/MRSH
	*Acinetobacter baumannii*	3	3/MBL(-)
	*Escherichia coli*	1	1/ESBL(-)
	*Enterobacter cloacae*	1	1/ESBL(-)
	*Enterococcus faecium*	1	1/VSE
**Central venous catheter-related infection**	*Staphylococcus haemolyticus*	1	1/MRSH
	*Enterococcus faecalis*	1	1/VSE
	*Acinetobacter baumannii*	1	1/MBL(-)
	*Klebsiella pneumoniae*	1	1/ESBL(+)
	*Escherichia coli*	1	1/ESBL(+)
**Intra-aortic balloon pump (IABP) infection**	*Staphylococcus epidermidis*	1	1/MSSE
**Urinary tract infection**	*Escherichia coli*	1	1/ESBL(+)

MRSE: methicillin-resistant *S. epidermidis*; MSSE: methicillin-susceptible *S. epidermidis; *MRSA: methicillin-resistant *S. aureus; *MSSA: methicillin-susceptible *S. aureus; *MRSH: methicillin-resistant *S. haemolyticus; *VSE: vancomycin-susceptible *Enterococcus*; ESBL: extended-spectrum β-lactamase; MBL: metallo-β-lactamase.

The association of the examined co-morbidities, intraoperative and postoperative factors and perioperative transfusions with development of microbiologically documented infection after cardiac surgery is presented in Table 6.

**Table 6:** Univariate logistic regression analysis of the association of various factors with development of infection.

Examined factors	With infection n = 24	Without infection n = 148	*p *Value
**Co-morbidities**			
Diabetes mellitus*	13 (22%)	46 (78%)	**0.031**
Arterial hypertension	18 (15.3%)	100 (84.7%)	0.469
COPD	4 (18.2%)	18 (81.8%)	0.542
Chronic renal failure	5 (45.5%)	6 (54.5%)	0.127
Peripheral vascular disease	5 (21.7%)	18 (78.3%)	0.253
Stroke	5 (26.3%)	14 (73.7%)	0.109
Unstable angina	6 (17.1%)	29 (82.9%)	0.543
Recent myocardial infarction	5 (10.9%)	41 (89.1%)	0.483
Older myocardial infarction	6 (19.4%)	25 (80.6%)	0.341
Pulmonary hypertension	1 (50%)	1 (50%)	0.195
Hyperlipidemia	7 (15.6%)	38 (84.4%)	0.718
Smoking	13 (18.8%)	56 (81.2%)	0.135
Alcohol abuse	1 (7.7%)	12 (92.3%)	0.506
**Intraoperative factors**			
Duration of anesthesia (hours)	6.78 ± 1.94	6.28 ± 1.65	0.181
Extra-corporeal circulation (min)	138.87 ± 85.88	119.27 ± 72.67	0.234
Ischemia time (min)	101,75 ± 61,76	88,95 ± 58,19	0.322
Degree of hypothermia (^°^C)	23.33 ± 12.40	25.40 ± 11.38	0.416
Emergency operation	5 (45.5%)	6 (54.5%)	0.545
**Postoperative factors**			
Intra-aortic balloon pump (IABP)	5 (27.8%)	13 (72.2%)	0.069
Duration of mechanical ventilation*	5.42 ± 10.31	1.34 ± 1.35	**0.014**
Re-intubation	11 (50%)	11 (50%)	0.337
Tracheotomy	7 (87.5%)	1 (12.5%)	0.133
Enteral nutrition for >10 days	5 (100%)	0	0.999
Parenteral nutrition for >10 days	6 (100%)	0	0.328
Re-operation	7 (53.8%)	6 (46.2%)	0.310
Complications in the CVICU*	19 (43.2%)	25 (56.8%)	**0.000**
Re-admission to the CVICU*	12 (60%)	8 (40%)	**0.000**
Total CVICU length of stay	12.54 ± 12.60	2.84 ± 2.60	0.519
CVICU length of stay >15 days	8 (80%)	2 (20%)	0.519
**Perioperative transfusions**			
N of RBC units transfused	6.79 ± 4.45	3.97 ± 2.35	**0.000**
N of FFP units transfused	7.00 ± 6.39	3.93 ± 2.96	**0.002**
N of PLT units transfused	8.13 ± 7.45	5.49 ± 4.57	**0.026**

*Variables significantly associated with infection (*p *value < 0.05).

COPD: Chronic obstructive pulmonary disease; CVICU: cardio-vascular intensive care unit

Multivariate analysis using a stepwise logistic regression analysis demonstrated that diabetes mellitus, the duration of mechanical ventilation, the presence of complications irrelevant to the infection during CVICU stay and CVICU re-admission are statistically significant factors for the development of a postoperative infection in cardiac surgical patients (Table 7).

**Table 7:** Statistically significant factors according to multiple logistic regression analysis associated with a higher risk to acquire infection.

Risk Factor	Odds ratio	Confidence Interval 95%	p Value
Diabetes mellitus	5.92	1.56-22.42	0.009
Duration of mechanical ventilation (days)	1.30	1.005-1.69	0.046
Complications in the CVICU	18.66	3.36-103.61	0.001
Re-admission to the CVICU	8.59	2.02-36.45	0.004

CVICU: cardio-vascular intensive care unit

Based on the regression analysis, the risk of acquiring an infection is 5.9 times greater in patients with diabetes mellitus compared to those without diabetes mellitus. Accordingly, for every day on mechanical ventilation the risk is increased by 30%, re-admission to the CVICU increases the risk by a factor of 8.6 and patients who presented other complications irrelevant to infection (cardiac, respiratory, renal, neurological, gastrointestinal, bleeding) in the CVICU had a 18.7 times higher risk of developing infection.

## Discussion

In this study, we found that the postoperative infection rate in patients undergoing on pump CABG surgery during their stay in our small volume, Cardio-Vascular Intensive Care Unit is 13.95%. This accounts for 24 patients out of 172 patients treated in this CVICU during the above mentioned time period. Six of these patients eventually died and infection was considered to be the primary cause of death. The observed mortality rate attributed to infection was 3.48% and the mortality rate among infected patients was 25%.

According to the logistic Euroscore scale, four out of the six patients who died (4/6 = 66%), were predicted to have a significantly increased risk of perioperative death (>6%). As we presented above, 39% of our patients were classified at Euroscore group III and IV and their combined, predicted mortality was 13%. All these patients required prolonged mechanical ventilation and increased length of stay in the CVICU, compared with the remaining patients of the study. The percentage of these patients in the study (39%) seems to be greater than in other studies and we attribute this to the special characteristics of our health region (isolated agricultural population with relatively restricted access to primary and advanced care facilities), their relatively increased age and the high percentage of co-morbidities.

Great variation exists in the literature about the rate of hospital infections among cardiac surgery patients. The ESGNI-008 (European Study Group on Hospital Infections) study [8] that took place in 42 hospitals in 13 European countries, found the overall prevalence of nosocomial infections in postoperative cardiac patients on the study day to be 26.8%. The Fowler et al. study [1] found that major infection occurred in 3.51% of patients and the associated mortality rate was 17.3%.

Sternotomy site infection was the most common infection in our study (8 patients, incidence 4.65%, 26.7% of all infections). Most of these patients were suffering from diabetes mellitus (75%) and two of them eventually died. The isolated microorganisms were *Staphylococcus epidermidis *(in 62.5% of cases), *Enterococcus faecium, Acinetobacter baumannii *and *Pseudomonas aeruginosa*. These rates are quite similar to those reported in the literature (infection rate: 0.4-9.7%, associated mortality rate: 7.2-14.2%, 90% of the cases due to *Staphylococcus *species) [9-11].

Central venous catheter-related infections were observed in our study too (incidence 2.9%, 16.7% of all infections). These findings were again in accordance with those reported in the literature. Although 4 of the patients who developed catheter-related infection died, the infection was not considered as the main cause of death as these patients presented other serious complications during their hospital stay. Michalopoulos et al. [5] described a rate of central venous catheter-related infection of 1.1% in the total number of patients (25.2% of those with infection), mainly due to gram-positive cocci.

Pneumonia was diagnosed and confirmed in 4 patients (incidence 2.32%, 16.7% of all infections) and 3 of them (75%) eventually died. The isolated microorganisms were *Acinetobacter baumannii *and *Pseudomonas aeruginosa*. These findings were significantly different from those observed by other studies where pneumonia was the main cause of postoperative infection in cardiac surgical patients and lower respiratory tract infections represented 57% of all infections [8]. However, the incidence of pneumonia in our study was in accordance with others where the incidence varies between 1.2% and 2.1% [12-14]. Hortal et al. [15] reported a high mortality rate (45.7%) and the most commonly isolated pathogens were gram-negative bacteria. We believe we cannot compare our results with other studies, as the total study population and the number of patients with infection were really small.

Mediastinitis was diagnosed in only one of our patients (incidence 0.58%, 3.3% of all infections) and the isolated microorganism was *Staphylococcus aureus *resistant to methicillin (MRSA). This patient died due to this infection. According to the literature data, the incidence of mediastinitis in cardiac surgery patients ranges from 0.6% to 2.65% [16-18] and the associated mortality varies between 14% and 23% [19,20].

The incidence of bacteremia in our study was 5.23%. The cases of bacteremia represented 30% of all infections (9/30). Four of these patients had pneumonia, one had a vascular catheter infection, one had an IABP infection and three of them had no other clinical site of infection. The isolated microorganisms in patients with bacteremia were gram-positive cocci and gram-negative bacteria (*Staphylococcus epidermidis, Staphylococcus haemolyticus, Acinetobacter baumannii, Escherichia coli, Enterobacter cloacae, Enterococcus faecium*). In a recent study [21], bacteremia was the second most common cause of clinical (after pneumonia). The cases of bacteremia represented 26.9% of all infections and 57.1% of them were not associated with any other identifiable infected site [21].

Finally, less common infections in our study were urinary tract infection (1 patient) and wound infection at the site of venous graft harvesting (1 patient).

Referring to co-morbidities, obesity [18,19], smoking [11,19], chronic obstructive pulmonary disease [18], arterial hypertension [21], and previous vascular surgery [21] have been previously reported as independent risk factors for the development of nosocomial infection. However, in our study, only diabetes mellitus is confirmed as an important risk factor of postoperative infection in cardiac surgery patients. Another study [22] found that the risk of infection and other serious life-threatening complications is 36% to 38% higher in diabetics and that insulin-treated diabetics had the highest risk of serious complications. In addition, it was observed that infection was a more common cause of death only in insulin-treated diabetics compared with patients without diabetes mellitus [22]. Furnary et al. [23] showed that increasing blood glucose levels were directly associated with increasing rates of death, deep sternal wound infections, length of hospital stay and hospital cost. They demonstrated that continuous insulin infusion therapy, designed to achieve predetermined target blood glucose levels, independently reduced the risks of death and deep sternal wound infections by 57% and 66%, respectively.

In the assessment of independent risk factors in our study, mechanical ventilation was proved to be an important factor that predisposes patients to infection. Our data show that for every day on mechanical ventilation, the risk is increased by 30%. This risk factor was also reported by other authors [24,25]. Patients treated on mechanical ventilation for more than 48 hours have a 5.4 times higher risk for developing severe postoperative infection, a 4 times higher risk for pneumonia and a 4.1 times increased risk for postoperative sepsis of unknown origin [26]. Mechanical ventilation promotes the introduction of colonized oropharyngeal contents into the lower respiratory tract mainly due to aspiration. Subsequent colonization of the lower airway is facilitated by debility, reduced consciousness, swallowing difficulty and defective host defense [27].

Re-admission to the CVICU is also a significant factor for infection. It is often difficult to discern whether complications that require re-admission are the consequence of an ongoing infection or they are irrelevant to the infection that develops after re-admission. Our patients presented 8.6 times higher risk for infection when re-admitted. The reasons for re-admission were respiratory (38.5%), cardiac (23.1%), cerebrovascular accident (15.4%), re-operation for a sternal wound debridement (15.4%), renal insufficiency requiring hemodialysis (3.8%) and seizure (3.8%). This is in accordance to other studies that also found that the most common reason for re-admission was respiratory failure, accounting for 47% of the patients [28]. In a recent study [29], preoperative renal failure, mechanical ventilation >24 hours, re-exploration for bleeding and low cardiac output state were independent predictors for re-admission to an intensive care unit. Chung et al. [30] observed that pneumonia was one of the most common complications developed during the second admission to the ICU. They concluded that ICU re-admission and its resultant extended ICU stay is a major morbidity outcome associated with high mortality and often prolonged ventilation, in addition to high economic cost [30].

An important finding in our study is that the development of postoperative non-infectious complications is associated with an increased risk of infection. Our study showed that patients who developed complications had 18.7 times higher chance of infection than those without complications. According to another study [31], the development of postoperative complications has been associated with an increased risk of death and prolonged hospital stay. It is quite interesting that this study showed that even though cardiac complications are more common, they are associated with less mortality and shorter ICU and hospital length of stay than non cardiac complications [31].

The relationship between the administration of blood derivatives and postoperative infections has been documented in patients undergoing cardiac surgery [26]. Although perioperative transfusions of RBC concentrates and other blood components commonly used in cardiac surgery patients, such as plasma and/or platelets, were implicated in our study in the development of postoperative infection in the univariate analysis (Table 6), it was not confirmed as an independent risk factor in the multivariate analysis (Table 7).

Furthermore, we did not find a significant relationship between age, ejection fraction or mode of surgery (elective or emergency) and postoperative infection in this study.

The main limitation of our study is the small number of the included patients. This is due to the low volume of patients treated at the Cardiothoracic surgery Department of our hospital. Additionally, the length of stay at the CVICU could not be evaluated as a risk factor for infection because of the small number of included patients and the scarce number of patients that stayed at the CVICU for >2 days. As a result, it was not possible to divide the patients into sub-groups according to the length of stay and examine whether the increased length of stay is associated with a higher rate of infection.

## Conclusions

Postoperative infections in cardiac surgery patients represent a serious problem and are directly related to patients' co-morbidities, intraoperative factors, as well as postoperative management. The risk of acquiring an infection is 5.9 times higher in patients with diabetes mellitus, is increased by 30% for every day on mechanical ventilation, is 8.6 times higher in patients re-admitted to the CVICU and is 18.7 times higher in patients who presented other complications, irrelevant to infection.

## Abbreviations

CVICU: Cardio-Vascular Intensive Care Unit; CABG: coronary artery bypass grafting; IABP: Intra-aortic balloon pump; pts: patients; MRSA: methicillin-resistant *S. aureus*; ESBL: extended-spectrum β-lactamase; MBL: metallo-β-lactamase; COPD: Chronic obstructive pulmonary disease.

## Competing interests

The authors declare that they have no competing interests.

## Authors' contributions

IL made substantial contribution to conception and design, was responsible for acquisition and analysis of data and wrote the manuscript. GP conceived of the study, and participated in its design, was involved in revising it critically for important intellectual content and gave final approval of the version to be published. AP was involved in drafting the manuscript and revising it critically. EA was involved in revising the manuscript critically and gave final approval of the version to be published. SL and HA gave final approval of the version to be published. All authors read and approved the final manuscript.
